# Comparative Transcriptome Analysis of Gonads for the Identification of Sex-Related Genes in Giant Freshwater Prawns (*Macrobrachium Rosenbergii*) Using RNA Sequencing

**DOI:** 10.3390/genes10121035

**Published:** 2019-12-11

**Authors:** Jianping Jiang, Xiang Yuan, Qingqing Qiu, Guanghua Huang, Qinyang Jiang, Penghui Fu, Yu Zhang, Yinhai Jia, Xiurong Yang, Hesheng Jiang

**Affiliations:** 1 College of Animal Science and Technology, Guangxi University, Nanning 530001, China; jiangjianping818@126.com (J.J.); cauyx2014@163.com (X.Y.); qiuscott@163.com (Q.Q.); jiangqinyang@126.com (Q.J.); fupenghui@sohu.com (P.F.); 456zhang16@sina.com (Y.Z.); 2 Guangxi Botanical Garden of Medicinal Plants, Nanning 530023, China; 3 Guangxi Academy of Fishery Sciences, Nanning 530021, China; gxnnhghua@sina.com; 4 College of Animal Science, Southwest University, Chongqing 402460, China; 5 Animal Husbandry Research Institute of Guangxi Zhuang Autonomous Region, Nanning 530001, China; yinhai18@163.com

**Keywords:** *Macrobrachium rosenbergii*, RNA-seq, gonad, sex-related candidate gene, SSR

## Abstract

The giant freshwater prawn (*Macrobrachium rosenbergii*) exhibits sex dimorphism between the male and female individuals. To date, the molecular mechanism governing gonadal development was unclear, and limited data were available on the gonad transcriptome of *M. rosenbergii*. Here, we conducted comprehensive gonadal transcriptomic analysis of female (ZW), super female (WW), and male (ZZ) *M. rosenbergii* for gene discovery. A total of 70.33 gigabases (Gb) of sequences were generated. There were 115,338 unigenes assembled with a mean size of 1196 base pair (bp) and N50 of 2195 bp. Alignment against the National Center for Biotechnology Information (NCBI) non-redundant nucleotide/protein sequence database (NR and NT), the Kyoto Encyclopedia of Genes and Genomes (KEGG) database, SwissProt database, Protein family (Pfam), Gene ontology (GO), and the eukaryotic orthologous group (KOG) database, 36,282 unigenes were annotated at least in one database. Comparative transcriptome analysis observed that 10,641, 16,903, and 3393 genes were significantly differentially expressed in ZW vs. ZZ, WW vs. ZZ, and WW vs. ZW samples, respectively. Enrichment analysis of differentially expressed genes (DEGs) resulted in 268, 153, and 42 significantly enriched GO terms, respectively, and a total of 56 significantly enriched KEGG pathways. Additionally, 23 putative sex-related genes, including *Gtsf1*, *IR*, *HSP21*, *MRPINK*, *Mrr*, and other potentially promising candidate genes were identified. Moreover, 56,241 simple sequence repeats (SSRs) were identified. Our findings provide a valuable archive for further functional analyses of sex-related genes and future discoveries of underlying molecular mechanisms of gonadal development and sex determination.

## 1. Introduction

The giant freshwater prawn, *Macrobrachium rosenbergii* (*M. rosenbergii*), as an important commercial freshwater prawn species, is widely distributed in China and Southeast Asian countries due to its delicious flesh and high value nutrition. Like other crustaceans, *M. rosenbergii* displays sexual dimorphic growth pattern: The male individuals grow much larger and faster than the females [1,2]. Thus, based on the economic impact, developing the monosex population culture as the efficient approach to boost production has received great attention from researchers in *M. rosenbergii*. Nevertheless, the premise of monosex culture is to understand the molecular mechanism of sex determination, as well as to unravel sex-related genes.

Studies on sex determination have proved that many macruran species exhibit the ZW/ZZ sex determination system [3,4,5]. In *M. rosenbergii*, sex reversal experiments proposed that the female prawns bear the ZW sex chromosomes, and the male individuals bear the ZZ chromosomes [1,6,7]. Subsequently, isolation of sex-specific markers further deepened the insight of the ZW/ZZ sex determination mode [8,9]. Recently, Ma et al. have confirmed that *M. rosenbergii* has the ZW/ZZ sex determination system based on the bacterial artificial chromosome (BAC) library, and nine genes were unraveled to be the key sex-linked genes by PacBio sequencing. Amongst, three genes, including zinc knuckle domain (ZKD), reverse transcriptase, and ANCDUO, protein homologs were known genes without characterization of roles in sex determination. Although the Z and W chromosomes have not been identified, the results still provided a useful resource and comprehensive knowledge of genomic structure of *M. rosenbergii* [10]. However, sex-related genes and a list of biological pathways involved in gonads development have been revealed [11,12,13,14,15,16], the potential molecular mechanisms of sex determination in *M. rosenbergii* are still not well understood.

Currently, along with the emergence of next generation sequencing (NGS), RNA-sequencing (RNA-seq) was widely applied to detect the expression profiles of genes and identify the sex-related genes in aquaculture species, especially those lacking genomic sequences, due to its high throughput and low cost. To date, some progresses have been made in the characterization of sex-related genes and pathways in *M. rosenbergii* [16], *Macrobrachium nipponense* [17], *Silver Sillago* [18], Olive Flounder [19], Pacific abalone [20], Pacific white shrimp [21], catfish [22], and *Acipenser dabryanus* [23]. Hence, the purpose of this study was to perform gonadal transcriptomic sequencing of females, super females, and males to investigate the gene expression profiles, and to identify the sex-related candidate genes. These data would lay the foundation for better illustrating the molecular mechanisms of sex determination.

## 2. Materials and Methods

### 2.1. Ethics Statement

All procedures were in compliance with the institutional guidelines and under a protocol approved by the Animal Experimental Ethical Inspection Form of Guangxi University (GXU2019-072).

### 2.2. Sample Collection 

The super female (WW genotype) individuals were purchased from the Enzootic Company from Israel (https://enzootic.com), and reared in a national *M. rosenbergii* seed multiplication farm in Nanning, Guangxi, China. The male (ZZ genotype) and female (ZW genotype) prawns from the same family were collected from this farm. For the WW individuals, they were achieved through a novel biotechnology with two steps: Females were sex-reversed into neo-males by injecting the suspended hypertrophied androgenic gland cells, crossing neo-males with normal females to validated WW females. Notably, the phenotypes of super females were not different from normal females and the fecundity of WW individuals has shown no significant deviation from the ZW individuals [24]. All the healthy experimental individuals used in this study were five months of age, and were maintained in aerated freshwater at 26 ± 2 °C in the previously mentioned farm. Ovaries from females (ZW) and super females (WW), and testes from males (ZZ), were collected and immediately frozen in liquid nitrogen, then stored at 80 °C until RNA extraction. For each of three groups, three biological replicates were used for RNA-seq, with each replicate being pooled by three prawns. The growth performance of the sample individuals is listed in Table 1 and Table S1.

### 2.3. RNA Extraction, Library Preparation, and Transcriptome Sequencing

Three individuals of each sample were pooled to extract the total RNA using Trizol Reagent (Invitrogen, Carlsbad, USA) according to the manufacturer’s instructions, and then treated with RNase-free DNase I (TianGen, Beijing, China) to eliminate genomic DNA contamination. The integrity and concentration of RNA was determined using the Agilent Bioanalyzer 2100 system (Agilent Technologies, California, USA) and the Qubit^®^ 2.0 Flurometer (Life Technologies, Carlsbad, USA), respectively. High-quality RNA samples (OD260/280 = 1.8–2.2, OD260/230 > 2.0, RNA integrity number (RIN) > 7.5, 28S: 18S > 1) was stored at −80 °C and used for library construction. A total of nine sequencing libraries were constructed with NEBNext^®^ UltraTM RNA Library Prep Kit for Illumina^®^ (NEB, Beijing, China) following the manufacturer’s recommendations. Finally, the libraries were sequenced with the Illumina Hiseq platform using paired-end strategy.

### 2.4. Transcriptome De Novo Assembly and Functional Annotation

The raw sequencing reads were processed through a Perl program for quality control. Clean reads were obtained by removing reads with adaptors and poly-N, removing ambiguous reads containing more than 10% unknown bases and the low-quality reads (the rate of reads in which the quality value ≤20 was more than 50%). Transcriptome assembly was accomplished by Trinity [25] with min_kmer_cov set to 2 by default and all other parameters set to default values. Then, a single set of nonredundant unigenes were acquired with the GICL clustering software [26].

Functional annotation of all assembled unigenes was performed with the following seven databases and four softwares: National Center for Biotechnology Information (NCBI) nonredundant nucleotide sequence database (Nt) based on the NCBI Blast (v2.2.28+) (https://blast.ncbi.nlm.nih.gov/Blast.cgi) (E-value < 1 × 10^−5^) NCBI nonredundant protein sequence database (Nr), SwissProt database, Kyoto Encyclopedia of Genes and Genomes (KEGG) (E-value < 1 × 10^−10^) using KEGG Automatic Annotation Server (KAAS) and eukaryotic orthologous group (KOG) database based on the diamond (version 0.8.22) (E-value < 1 × 10^−3^), Protein family (Pfam) by Hmmscan with the E-value less than 0.01, and Gene Ontology (GO) through Blast2GO (version 3.0) (https://www.blast2go.com/) (E-value < 1 × 10^−6^), respectively. The unigenes annotated by at least one database were considered as annotated successfully based on the corresponding threshold. When unigenes were different in the cases where searches against different databases disagreed with each other, the desired annotation information was according to our experiment objective and the reliability of different databases (NR > GO > KEGG > SwissProt > Pfam > KOG > NT). Thus, in our study, we preferred the annotated results of NR database.

### 2.5. Identification of Differentially Expressed Genes (DEGs)

RSEM software (version v1.2.15) was used for reads mapping to the assembled reference transcriptome with default parameters [27]. Gene expression levels were calculated based on the expected number of fragments per kilobase of transcript sequence per millions base pairs sequenced (FPKM). Differential gene expression analysis among the three groups was performed with the DESeq2 [28] within *q*-value < 0.01, |log2 (fold change)| > 2. Additionally, GO enrichment and KEGG pathway analysis of DEGs was implemented with the GOseq tool and online software KOBAS (Version 3.0) (http://kobas.cbi.pku.edu.cn/index.php) [29,30], respectively. Among them, those with *p*-value < 0.05 were considered to be significantly enriched.

### 2.6. Detection of Simple Sequence Repeats (SSRs)

MIcroSAtellite (MISA) software (http://pgrc.ipk-gatersleben.de/misa/misa.html) was used to identify the simple sequence repeats (SSRs) of the transcriptome. Six types of SSR were investigated, including mono-, di-, tri-, tetra-, penta-, and hexa-nucleotide motifs. The Primer 3 software (http://primer3.sourceforge.net/releases.php) was then used for primer design based on the flanking regions of SSRs for further validation.

### 2.7. Real-Time Quantitative Reverse Transcription PCR (qRT-PCR) 

To validate the accuracy of gene expression data obtained by RNA-seq, 10 DEGs were selected to be verified by qRT-PCR using the same samples for RNA-seq. The 10 selected DEGs consisted of 6 sex-related candidate genes (ubiquitin carboxyl-terminal hydrolase 46, transcription factor SOX-10-like, heat shock protein 70, heat shock 70 kDa protein 1A/1B, male reproductive-related protein B, and male reproductive-related serum amyloid A), one DEG (heat shock protein 10) from the heat shock protein family, one DEG (slow-type skeletal muscle actin 4) with the highest expression in ZZ gonads, and the two DEGs (neuronal-specific septin-3 and tubulin α-1 chain-like) with two-fold different expression between WW gonads and ZW gonads. PCR Primers were designed with Primer 3 and Oligo 7.0 (Table S2). Then *18S* was used as a housekeeping gene to normalize the mRNA levels of DEGs [31,32]. The qRT-PCR was performed in triplicate by using the Lightcycler 480 II (Roche Applied Science, Penzberg, Germany) with the following reaction conditions: Pre-denaturation at 95 °C for 10 s; amplification 45 cycles of 95 °C for 10 s, 59 °C for 10 s, and 72 °C for 10 s, and using the following program: 95 °C for 10 min; 45 cycles of 95 °C for 10 s, 60 °C for 10 s, and 72 °C for 10 s; 72 °C for 6 min. The relative expression levels were calculated with the method of 2^−ΔΔCt^ as described previously [33].

## 3. Results

### 3.1. Sequencing Analysis and Transcriptome Assembly

**Figure 1:**
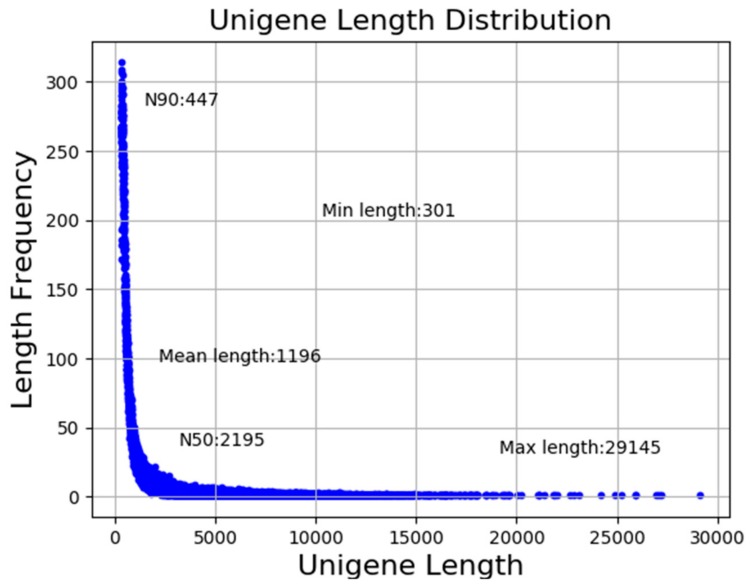
Length distribution of unigenes.

In the present study, a total of 70.33 gigabases (Gb) of clean bases were generated. The clean reads ranging from 47,066,162 to 60,480,354 for nine libraries with the GC% of approximately 42% per library were used for further analysis. In addition, Q20 and Q30 of each library were higher than 97% and 93%, respectively (Table 2). As illustrated in Figure 1, a total of 115,338 unigenes were assembled with a mean size of 1196 bp and N50 of 2195 bp.

### 3.2. Sequence Annotation

All unigenes were searched against the Nr, Nt, KEGG, SwissProt, Pfam, GO, and KOG database, among them, most of the unigenes were annotated in Pfam and GO databases, while 2543 unigenes were annotated in all seven databases. Meanwhile, 36,282 unigenes founded as putative homologues were annotated at least in one database. The annotation success rate of unigenes was 23,483 in Nr (20.36%), 6556 in Nt (5.68%), 9390 in KEGG (8.14%), 15,315 in SwissProt (13.27%), 27,160 in Pfam (23.54%), 27,160 in GO (23.54%), and 8158 in KOG (7.07%) (Table 3).

**Figure 2:**
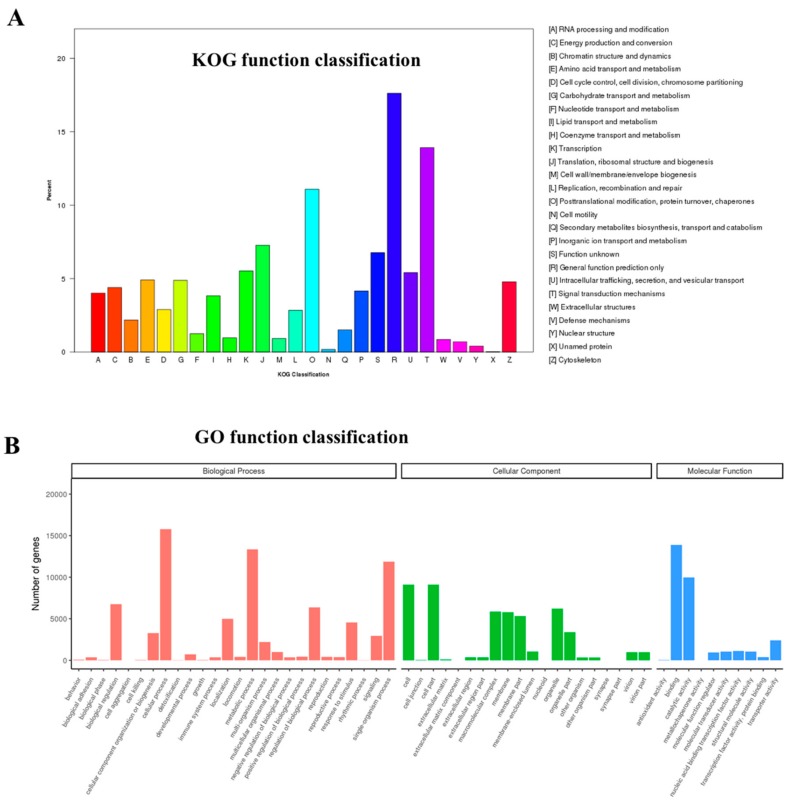
Functional classifications of unigenes in *M. rosenbergii* gonadal transcriptome. (**A**) eukaryotic orthologous group (KOG) classification. (**B**) Gene Ontonogy (GO) classification.

Through the KOG function classification, 8158 unigenes were classified into 26 functional categories, the top 3 of which were as follows: General function prediction only (1437 unigenes), signal transduction mechanisms (1135 unigenes), and posttranslational modification, protein turnover, chaperones (904 unigenes) (Figure 2A).

Aligning to the GO database, a total of 27,160 unigenes were sorted into three major GO categories: Biological processes, molecular functions, and cellular components. Among them, “cellular process” (15,773), “cell part” (9096), and “cell” (9096), as well as “binding” (13,890), were the main terms, respectively (Figure 2B).

As for KEGG assignment, 9390 unigenes were mapped to 230 KEGG pathways, 4.12% (387 unigenes) of which were mapped to “Ribosome”, followed by “Lysosome”, and “Phagosome”. In addition, we identified several sex-related KEGG pathways, such as MAPK signaling pathway (ko04010, 134 unigenes), GnRH signaling pathway (ko04912, 75 unigenes), progesterone-mediated oocyte maturation (ko04914, 77 unigenes), focal adhesion (ko04510, 176 unigenes), calcium signaling pathway (ko04020, 130 unigenes), ubiquitin mediated proteolysis (ko04120, 105 unigenes), and wnt signaling pathway (ko04310, 81 unigenes) (Table S3).

### 3.3. DEGs Identification and Enrichment Analysis

**Figure 3:**
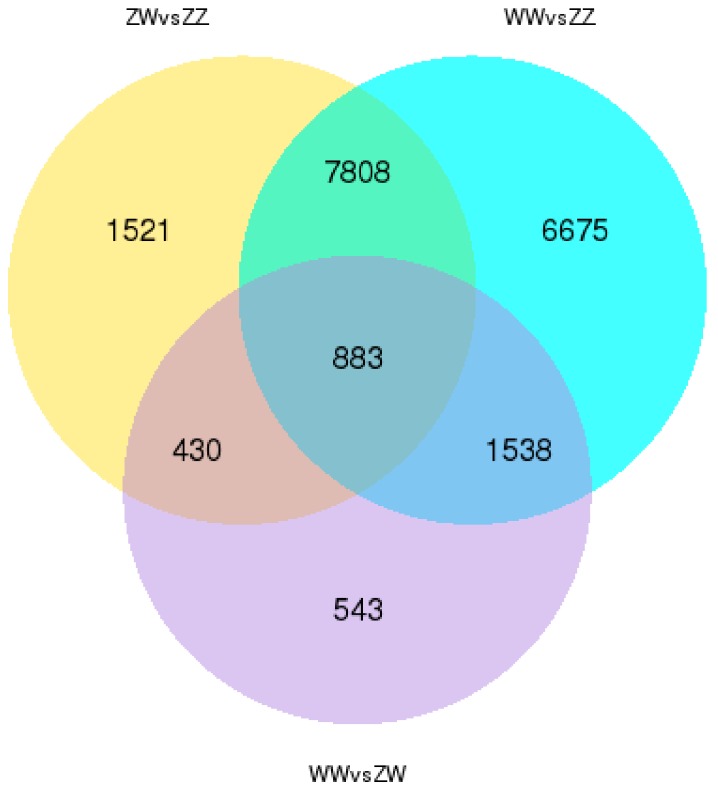
Venn diagram showing the numbers of expressed genes and differentially expressed genes (DEGs) in ZW, WW, and ZZ.

As a result, 10,641 genes were significantly differentially expressed in ZW vs. ZZ and 16,903 genes were found differentially expressed in WW vs. ZZ. Between WW vs. ZW, only 3393 genes were revealed as DEGs (Figure 3 and Table S4).

**Figure 4:**
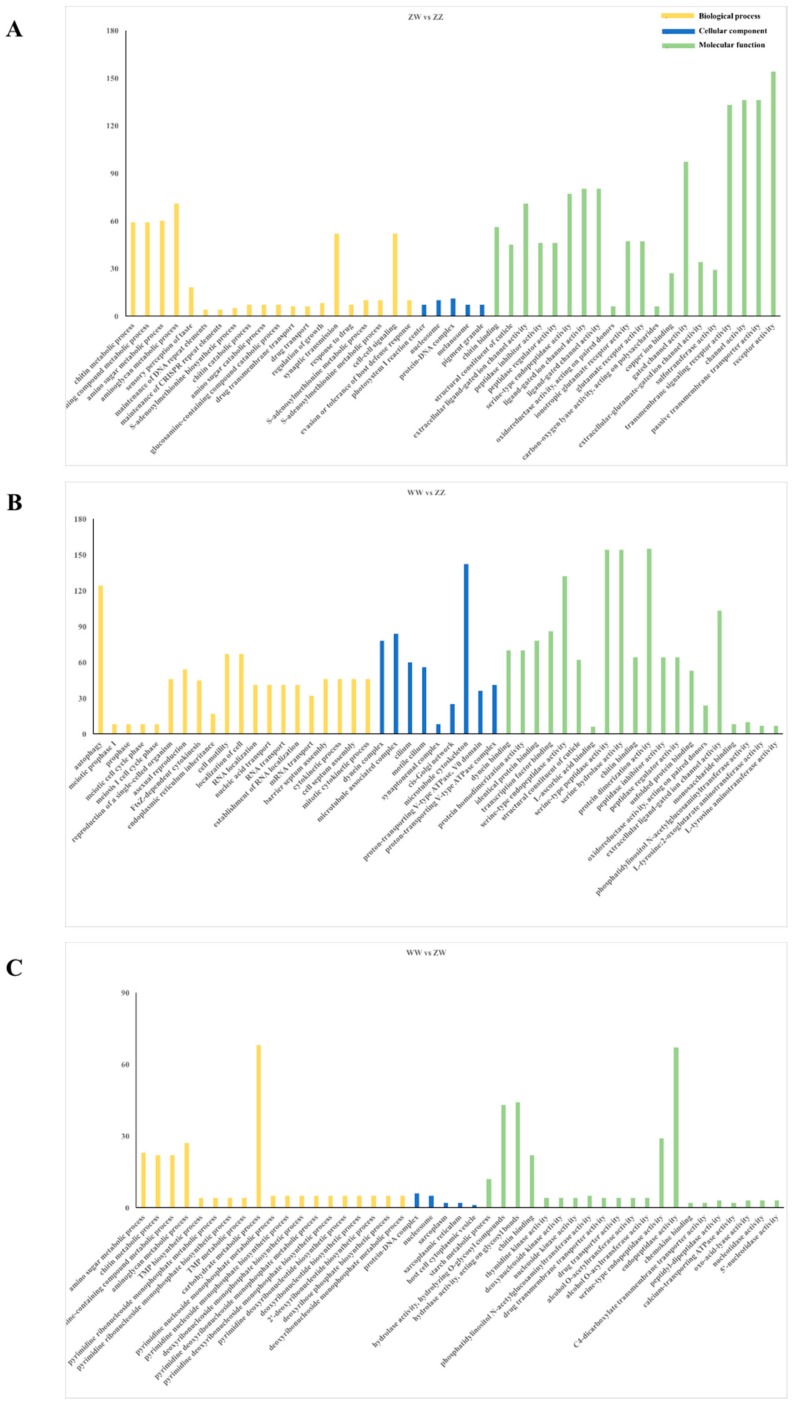
GO enrichment analysis of DEGs. (**A**) GO enrichment analysis of DEGs in ZW vs. ZZ. (**B**) GO enrichment analysis of DEGs in WW vs. ZZ. (**C**) GO enrichment analysis of DEGs in WW vs. ZW.

GO annotation was performed to classify the DEGs among the three groups. As a result, 268, 153, and 42 GO terms were significantly enriched, respectively (Figure 4). Amongst them, the most enriched GO terms at the level of biological processes were “chitin metabolic process” and “glucosamine-containing compound metabolic process”. At the molecular function level, “dynein binding” was the dominant term. At the cellular component, “dynein complex” was the most significantly enriched term.

**Figure 5:**
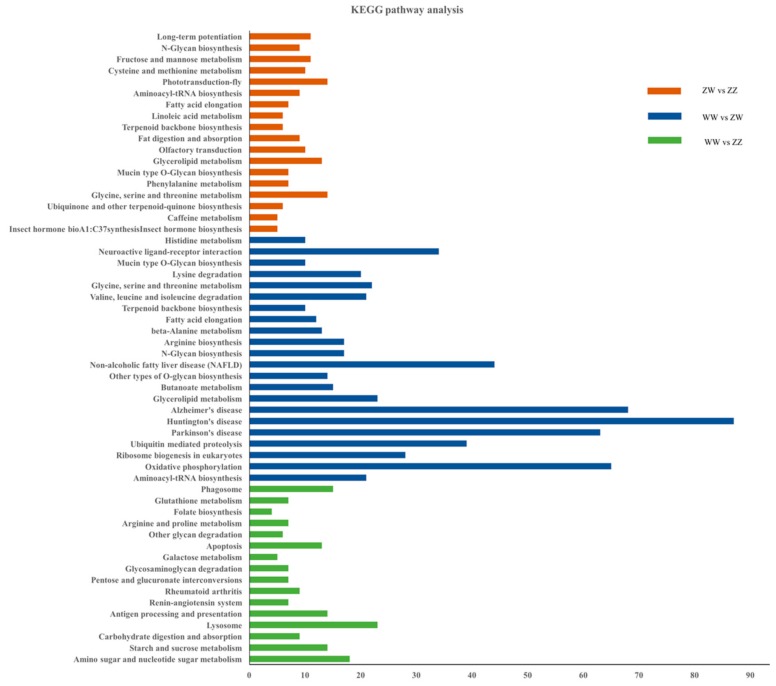
Kyoto Encyclopedia of Genes and Genomes (KEGG) pathway analyses of DEGs.

Meanwhile, enrichment analysis of KEGG pathway was conducted. Totally, 56 KEGG pathways were significantly enriched. The top two signaling pathways were “amino sugar and nucleotide sugar metabolism” and “starch and sucrose metabolism” (Figure 5).

### 3.4. Genes of Interest Related to Sex

Based on the functional annotation of unigenes, along with the previous publications, 23 sex-related candidate genes implicated in gonadal development and sex determination were identified (Table 4). Notably, among them, two genes (gametocyte-specific factor 1 and insulin-like receptor) showed specific expression in ovaries between female and super female. And seven genes, including heat shock protein 21, heat shock protein isoform 12Ai1, male reproductive tract-specific Kazal-type proteinase inhibitor, male reproductive-related protein, male reproductive-related protein B, male reproductive-related protein A, and male reproductive-related protein Mar-Mrr, showed male-specific expression patterns. These genes could be considered as the most promising sex-related candidates.

### 3.5. Discovery of Simple Sequence Repeats (SSRs) 

**Figure 6:**
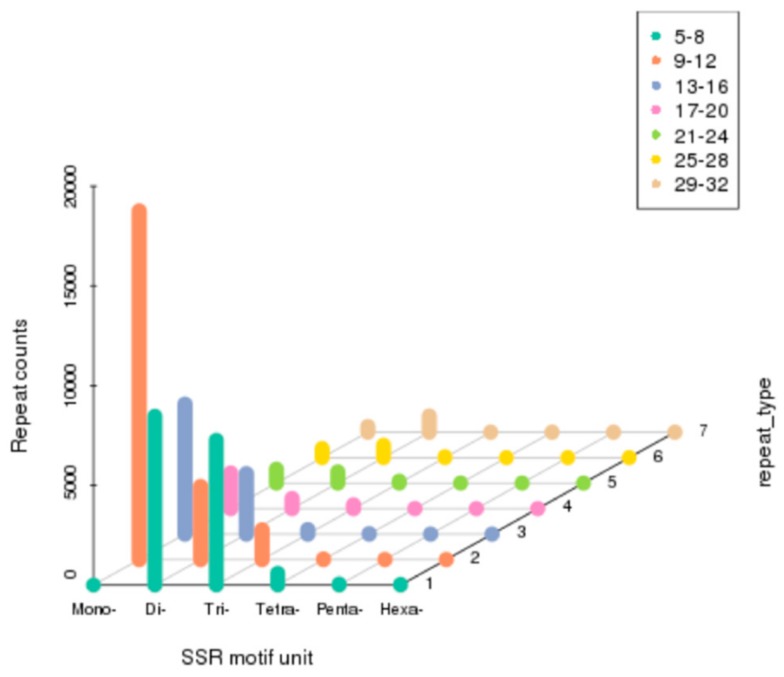
Distribution of identified simple sequence repeats (SSRs) according to motif types in the sequences of *M. rosenbergii.*

In the present study, MicroSAtellite (MISA) software was used to detect the SSRs of *M. rosenbergii.* As a result, a total of 56,241 SSRs were identified in 33,189 SSR-containing sequences (Figure 6). Among them, mononucleotide (27,574, 49.03%) was the dominant, followed by dinucleotide (18,598, 33.07%), trinucleotide (9311, 16.56%), tetranucleotide (655, 1.16%), pentanucleotide (63, 0.11%), and hexanucleotide (40, 0.07%).

### 3.6. The qRT-PCR Validation

**Figure 7:**
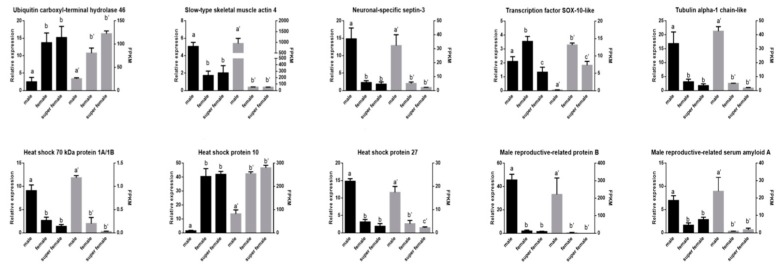
Verification of the expression patterns both in real-time quantitative reverse transcription PCR and RNA-seq. Groups with different letters were significantly different (*p* < 0.05).

Furthermore, 10 DEGs were selected for qRT-PCR validation. As a result, all the detected genes showed similar expression patterns (Figure 7), which indicated the reliability and accuracy of our transcriptome analysis.

## 4. Discussion

As an economically important aquaculture species, a better understanding of the genetic and biological mechanisms underlying the complex ZW/ZZ sex determination system of *M. rosenbergii* is important, yet it is poorly elucidated. Sagi et al. firstly reported the sex dimorphism in *M. rosenbergii* [34]. Subsequently, two sex reversal experiments uncovered that *M. rosenbergii* bears the ZW/ZZ sex determination system. Mating of sex-reversed females (neo-males) with normal females (ZW) could increase a higher ratio of females, while mating of sex-reversed males (neo-females) with normal males could produce all-male (ZZ × ZZ) progeny [1,6,7]. Recently, along with the emergence of high-throughput sequencing, RNA-seq as a most powerful tool is available for illustrating the mechanisms of immune response [35], determining the expression pattern of reproduction, growth, and pheromone communication in hepatopancreas, gill, muscle, and antennal gland [36,37,38]. In the present study, we profiled to detect the transcriptomes of gonads in females, super females, and the males using RNA-seq, with the aim of interpreting the molecular mechanism involved in the sex determination, and identifying sex-related candidate genes.

The general consensus in the prawn (*M. rosenbergii*) culture industry is that the major approach to potentially boost the prawn production is to produce all females, due to their normal size distribution, higher production, and product values compared to all males [2]. Hence, in our study, to better illustrate the molecular mechanism of sex-determination, we used the normal females, super females, and males to detect the gonadal expression profile. Therein the male individuals and normal female individuals were from one full-sib family, and, along with the super females, they were reared in the same conditions and environments in the national *M. rosenbergii* seed multiplication farm. Moreover, all the healthy samples were five months; meanwhile, the body weight and body length of samples, as well as the development of ovaries for females and super females, were similar, thus reducing the background effects as far as possible.

We identified 23 sex-related candidate genes. Among them, two and seven genes showed specific expression patterns in ovaries and testes. Gametocyte-specific factor 1 (*Gtsf1*) as a member of unknown protein family 0224 (UPF0224) was involved in Piwi-interacting RNA (piRNA) pathway [39]. Some evidences indicated that *Gtsf1* participated in spermatogenesis, and had significant impact on male germ cells [39,40]. Moreover, in teleost, *Gtsf1* as a key candidate gene was involved in sex differentiation [41,42]. Intriguingly, in this study, *Gtsf1* displayed sexual dimorphic expression and no expression was detected in testes. Furthermore, the expression in super females was more than two-fold higher than that in females. The current data insinuated that *Gtsf1* could be considered as a key candidate gene related to gonadal development and sex determination.

Gonadotropin-releasing hormone II receptor (*GnRHR2*) was originally identified in humans [43]. Evidence has been accumulated that *GnRH2* is required for sexual behavior [41]. Furthermore, initial studies have discovered that GnRHR2 together with its ligand GnRH2, were considered to be novel regulators in driving the reproduction process in mammals [44,45]. Toward this end, our data detected that *GnRHR2* was upregulated in ovaries. Taken together, we conjectured that *GnRHR2* has essential functions in female reproduction.

Insulin-like receptor (*IR*) functions as the pivotal member of insulin family signaling pathway and directs the male sexual differentiation in mammals [46]. Earlier evidence has shown that *IR* interacts with the insulin-like androgenic gland hormone (*IAG*) to regulate sex differentiation and spermatogenesis in crustaceans [13,15,46,47]. In our study, *IR* has shown higher expression in ovaries than in testes. Notably, the expression of *IR* in super females was more than two-fold than that in females. The results presented here suggest that *IR* could be a promising candidate gene for sex differentiation between females and super females.

Sex determination protein fruitless-like (*Fru*) was specially displayed in regulating the sexual orientation and sex behavior in *Drosophila melanogaster* [48,49]. In our transcriptome database, *Fru* showed sexual dimorphism in ovaries and testes, which displayed upregulated expression in ovaries. As observed in the Chinese mitten crab [50], *Fru* was implicated in sex determination pathway-like in *Drosophila melanogaster*.

Spermatogenesis-associated protein 5-like (*SPATA5L*) belonging to the ATPase family protein 2 homolog was first identified in mice and has essential functions in spermatogenesis, alopecia areata, intellectual disability, and other serious disorders [50]. Ge et al. have shown that down regulation of the expression of *SPATA5L* in males could decrease the fecundity of females during mating [51]. In our research, *SPATA5L* was expressed at higher levels in ovaries. Taken together, the current data provides a hint that *SPATA5L* might be a promising candidate for sex differentiation.

Transcription factor SOX-10-like as a member of SRY-related transcription factors from the Sox (Sry-type HMG box) E subfamily was reported to play a critical role in testis development [18,52]. In our research, it was expressed higher in ovaries than that in testes.

The ubiquitin-related homologous genes have been investigated and shown higher expression in testis and ovary, and further involved in reproductive process [53]. In our gonadal transcriptome database, two ubiquitin-related homologous genes (ubiquitin carboxyl-terminal hydrolase 46 and ubiquitin carboxyl-terminal hydrolase isozyme L5) were identified. It is important to further characterize these genes in *M. rosenbergii* to illustrate their potential role in sex differentiation and sex determination.

Cytochrome P450 302A1 (*CYP302a1*) was a new member of the cytochrome P450 (CYP) super-family. It encodes 22-hydroxylase and participates in ecdysteroid biosynthesis [54,55]. In drosophila and mosquito, *CYP302a1* was highly expressed in females, which further suggested that *CYP302a1* has an important role in the ovaries, as described by Chavez et al. [56] and Warren et al. [54]. Thus, our data have shown that *CYP302a1* was also expressed predominantly in the ovaries, which was in accordance with previous studies and further supporting our finding of the function of *CYP302a1* in the ovary.

Cytochrome P450 CYP315a1 (*CYP315a1*), another a member of the CYP super-family gene, was reported to be implicated in the ecdysteroidogenic pathway in *Bombyx mori* [57] and *Drosophila melanogaster* [54]. As we all know, steroid hormones were responsible for controlling reproduction and development in higher organisms and arthropods [58]. Thus, we deduced that *CYP315a1* may be involved in testicular development.

Forkhead box l2 (*Foxl2*), a member of Fox gene family, encodes a conserved transcription factor and is a special marker of ovarian differentiation [59]. Moreover, *Foxl2* plays a critical role in ovarian differentiation and maintenance [60]. In our study, interestingly, *Foxl2* displayed upregulated expression in testes, and has shown similar expression pattern in *Litopenaeus vannamei* [21]. However, the corresponding role in *M. rosenbergii* remains to be determined.

Heat shock proteins (HSPs) contribute to the interaction with steroid hormone receptors, temperature, and estrogen signaling, etc. [61,62]. Furthermore, HSPs were considered as promising candidates with potential effects on temperature-dependent sex determination (TSD) [62]. In addition, Matsumoto et al. have verified that heat shock cognate 70 kDa proteins played a regulatory role in mouse spermatogenesis [63]. In the present study, all three HSPs (heat shock protein 27, heat shock protein 70, and heat shock protein 70 cognate3) exhibited upregulated expression pattern in the testes, while heat shock 70 kDa protein 1A/1B, heat shock protein 21, and heat shock protein isoform 12Ai1 displayed testis-specific expression patterns as well.

Some male reproductive-related genes were also identified. Among them, two male-biased genes (male reproductive-related protein B and male reproductive-related serum amyloid A) and two testis-specific genes (male reproductive-related protein and male reproductive-related protein A) were upregulated and showed a specific expression pattern in testes which was similar to the results of Dai et al. [64]. Meanwhile, the other two testis-specific genes, including male reproductive-related protein Mar-Mrr and male reproductive tract-specific Kazal-type proteinase inhibitor, showed a specific testicular expression pattern. Earlier studies had demonstrated that *Mar*-*Mrr* was specially expressed in the male reproductive tract, and was required for sperm function [11,14,65]. Thus, combining the current results indicates that *Mar*-*Mrr* is implicated in male-reproduction.

Peptidase inhibitors were not only functional on cell migration, signal transmission, wound healing, and tissue remodeling [66], but also on male-reproduction in *Drosophila* [67]. Previous studies have confirmed that the male reproduction-related peptidase inhibitor Kazal-type (*MRPINK*) gene has a potential effect on male reproductive processes in prawns and crab [12,68,69]. In our study, one peptidase inhibitor (male reproductive tract-specific Kazal-type proteinase inhibitor) was expressed at higher levels in male gonads, suggesting that the peptidase inhibitor plays a critical role in gonadal development.

Of note, these identified sex-related genes and RNA-seq data could promote our understanding on the molecular mechanism involved in the sex determination. In addition, RNA interference (RNAi) and clustered regularly interspaced short palindromic repeats/CRISPR associated (CRISPR/Cas9)-mediated genome editing has revolutionized the gene functional determination, and was applied to controlling of sex development in aquaculture species [15,70,71]. Definitely, the actual functional roles of these selected sex-related genes were urgently required to be investigated, and the sex-specific genes may provide strong support for further sex manipulation and monosex culture based on the genome editing strategy.

Notably, numerous SSRs were identified. Currently, SSRs, single nucleotide polymorphisms (SNPs), and insertions and deletions (indels) are widely used for genetic linkage mapping, quantitative trait locus (QTL) detection, genetic diversity assessment etc. In *M. rosenbergii*, See et al. have developed microsatellite markers to evaluate genetic diversity [72]. Using the high throughput sequencing, it is feasible to detect and discover the large numbers of SSRs [37,73]. Correspondingly, the SSRs identified in the present study could serve as genetic markers for further QTL mapping and marker-assisted selection (MAS) in *M. rosenbergii*.

## 5. Conclusions

This is the first comprehensive report on the gonadal transcriptome of the *M. rosenbergii*, and 115,338 unigenes were identified. By comparing ovary and testis transcriptomes, numerous DEGs were identified, and 23 sex-related genes were revealed. Moreover, 56,241 SSRs were discovered and could be used as genetic markers. These findings established a valuable database for future functional analyses of sex-related genes and understanding the molecular mechanisms of sex determination in *M. rosenbergii*.

## Figures and Tables

**Table 1:** Growth performance of the 27 samples among the three groups.

Sample	Body Weight (g)	Body Length (cm)
ZW	9.61 ± 0.44	6.31 ± 0.16
WW	9.65 ± 0.28	7.62 ± 0.12
ZZ	13.09 ± 0.32	7.02 ± 0.11

**Table 2:** Summary statistics of gonadal transcriptome sequencing data for *Macrobrachium rosenbergii* (*M. rosenbergii*).

Sample	Raw Reads	Clean Reads	Clean Bases	Q20 (%)	Q30 (%)	GC (%)
ZW_1	52,214,594	47,435,802	7.12G	97.84	93.90	42.00
ZW_2	57,714,006	52,410,538	7.86G	97.81	93.82	41.88
ZW_3	52,276,492	47,066,162	7.06G	97.77	93.74	41.75
WW_1	60,924,940	54,729,552	8.21G	98.04	94.31	41.81
WW_2	62,970,576	57,154,534	8.57G	97.99	94.18	41.85
WW_3	66,416,082	60,480,354	9.07G	97.94	94.10	41.69
ZZ_1	54,655,372	49,757,368	7.46G	97.68	93.52	41.85
ZZ_2	57,466,470	52,158,488	7.82G	97.71	93.62	41.93
ZZ_3	52,503,322	47,730,328	7.16G	97.91	94.07	43.53

**Table 3:** Statistics of annotation results.

Database	Number of Unigenes	Percentage (%)
Nr	23,483	20.36
Nt	6,556	5.68
KEGG	9,390	8.14
SwissProt	15,315	13.27
Pfam	27,160	23.54
GO	27,160	23.54
KOG	8,158	7.07
all database	2,543	2.20
at least one database	36,282	31.45

**Table 4:** List of sex-related candidate genes in *M. rosenbergii.*

Gene Description	Hit Species	Mean FPKM (ZW)	Mean FPKM (WW)	Mean FPKM (ZZ)	NR ID	NR E-Value
gametocyte-specific factor 1	*Macrobrachium nipponense*	3.58	7.12	0.02	AMY62701.1	2.20 × 10^−6^
gonadotropin-releasing hormone II receptor	*Orussus abietinus*	0.87	0.71	0.16	XP_012272108.1	8.8 × 10^−95^
insulin-like receptor	*Trachymyrmex zeteki*	2.35	16.93	0.00	KYQ55071.1	4.60 × 10^−183^
PREDICTED: sex determination protein fruitless-like	*Fopius arisanus*	11.21	6.76	0.99	XP_011299024.1	3.70 × 10^−31^
spermatogenesis-associated protein 5-like	*Planoprotostelium fungivorum*	7.12	16.61	2.50	PRP83878.1	3.30 × 10^−56^
transcription factor SOX-10-like	*Zootermopsis nevadensis*	13.15	7.33	0.21	XP_021918795.1	1.60 × 10^−49^
ubiquitin carboxyl-terminal hydrolase 46	*Aedes aegypti*	80.51	122.33	25.60	XP_021706106.1	3.70 × 10^−147^
Ubiquitin carboxyl-terminal hydrolase isozyme L5	*Scylla paramamosain*	24.24	77.3	26.76	ACM43511.1	6.60 × 10^−150^
cytochrome P450 CYP302a1	*Portunus trituberculatus*	249.96	271.16	0.86	AJA06113.1	3.80 × 10^−152^
cytochrome P450 CYP315a1	*Portunus trituberculatus*	2.61	1.25	83.75	AJF94636.1	5.00 × 10^−147^
forkhead box L2	*Procambarus clarkii*	6.64	1.83	29.28	ALD48735.1	3.70 × 10^−99^
heat shock protein 27	*Procambarus clarkii*	3.93	2.21	17.4	AGZ84434.1	7.60 × 10^−53^
heat shock protein 70	*Portunus trituberculatus*	2.02	0.23	10.24	ACZ02405.1	5.00 × 10^−308^
heat shock protein 70 cognate 3	*Eriocheir sinensis*	0.67	0.04	2.51	AHA61465.1	8.30 × 10^−300^
heat shock 70 kDa protein 1A/1B	*Toxocara canis*	0.20	0.02	1.18	KHN76385.1	8.60 × 10^−153^
heat shock protein 21	*M. rosenbergi*	0.11	0.10	2.05	AET34915.1	8.80 × 10^−31^
heat shock protein isoform 12Ai1	*Cherax destructor*	0.10	0.05	0.95	AKB96220.1	2.40 × 10^−97^
male reproductive-related protein B	*M. rosenbergi*	1.91	0.01	221.43	ABQ41229.1	1.00 × 10^−32^
male reproductive-related protein	*M. rosenbergi*	0.00	0.00	8.97	ABQ41231.1	3.60 × 10^−40^
male reproductive-related serum amyloid A	*M. rosenbergi*	0.96	1.76	23.86	ABQ41247.1	2.40 × 10^−39^
male reproductive-related protein A	*M. rosenbergi*	0.00	0.00	3.04	ABQ41213.1	6.80 × 10^−22^
male reproductive tract-specific Kazal-type proteinase inhibitor	*M. rosenbergi*	0.20	0.00	195.26	AAX83134.1	8.40 × 10^−76^
male reproductive-related protein Mar-Mrr	*M. rosenbergi*	0.33	0.00	120.44	ABQ41239.1	8.20 × 10^−32^

## Supplementary Materials

The following are available online at https://www.mdpi.com/2073-4425/10/12/1035/s1: Table S1: Growth performance of the 27 samples among the three groups. Table S2: Primers of the 10 DEGs used for qRT-PCR. Table S3 KEGG pathway analysis of 9,390 unigenes. Table S4 DEGs in ZW vs. ZZ, WW vs. ZZ, and WW vs. ZW.
